# Processing of Emotion in Functional Neurological Disorder

**DOI:** 10.3389/fpsyt.2018.00479

**Published:** 2018-10-05

**Authors:** Petr Sojka, Martin Bareš, Tomáš Kašpárek, Miroslav Světlák

**Affiliations:** ^1^Department of Neurology, Faculty of Medicine, Masaryk University and St Anne's University Hospital Brno, Brno, Czechia; ^2^Department of Psychiatry, Faculty of Medicine, Masaryk University and University Hospital Brno, Brno, Czechia; ^3^Department of Psychology and Psychosomatics, Faculty of Medicine, Masaryk University and University Hospital Brno, Brno, Czechia

**Keywords:** functional neurological disorder, interoception, emotion, emotional abuse, predictive coding

## Abstract

Emotions have traditionally been considered crucial in the development of functional neurological disorder, but the evidence underpinning this association is not clear. We aimed to summarize evidence for association between functional neurological disorder and emotions as formulated by Breuer and Freud in their conception of hysterical conversion. Based on a systematic literature search, we identified 34 controlled studies and categorized them into four groups: (i) autonomic arousal, (ii) emotion-motion interactions, (iii) social modulation of symptoms, and (iv) bodily awareness in FND. We found evidence for autonomic dysregulation in FND; convergent neuroimaging findings implicate abnormal limbic-motor interactions in response to emotional stimuli in FND. Our results do not provide enough empirical evidence for social modulation of the symptoms, but there is a clinical support for the role of suggestion and placebo in FND. Our results provide evidence for abnormal bodily awareness in FND. Based on these findings, we propose that functional neurological symptoms are forms of emotional reactions shaped into symptoms by previous experience with illness and possibly reinforced by actual social contexts. Additional research should investigate the effect of social context on the intensity of functional neurological symptoms and associated brain regions.

## Introduction

Functional neurological disorders (FND) refer to patients with neurological symptoms in the absence of neurological disease. These symptoms have also been labeled as “hysterical,” “psychogenic,” “non-organic,” or “medically unexplained.” FND are common in neurology wards with the levels of disability similar to epilepsy or multiple sclerosis (1). Despite the long-standing interest in FND and the growing body of neuroimaging research in the last decade, the etiology of FND remains elusive.

Several etiological models of the disorder have been proposed throughout the history with varying degree of suggested psychological function of the symptoms (2). Whereas in the original formulation of hysterical conversion by Breuer and Freud (3) and in the following psychodynamic theories of FND (4, 5), psychological factors, notably emotions, played a crucial role in the etiology of functional neurological disorders, cognitive and later neurobiological models emphasized other than emotional factors and framed FND as defense behaviors (6, 7), and attention and expectation abnormalities (8).

Some authors question the importance of emotions in the etiology of the disorder, because psychological stressors and emotional dysregulation are not always apparent in FND (8). The change in the clinical focus can be seen in the current revision of DSM-V where association of motor or sensory symptoms with psychological cause has been left out from diagnostic criteria for the disorder (as also reflected in the name change from “psychogenic” to “functional neurological disorder”; APA (9). However, recent experimental studies provide some evidence for abnormal limbic-motor interactions in FND and point to the relevance of emotions in the etiology of the disorder.

In this review, we aim to summarize evidence for the hypothesis that functional neurological symptoms are emotional expressions of distress. We build on the original idea of connection between emotions and physical symptoms formulated in *Studies on Hysteria*. In their seminal work, Breuer and Freud (3) suggest that “the excitation arising from the affective idea is ‘converted' into a somatic phenomenon” (p.206). The authors claim that FND patients have higher levels of affective (cerebral) excitation and that ideation has greater effect on nervous apparatuses of organs in FND. Following Pierre Janet, the authors of *Studies on Hysteria* also document altered bodily awareness in conversion hysteria.

To address the conversion hypothesis in a manageable way, we break it into the following four questions: (1) Is there evidence for higher sensitivity to emotional distress in FND? (2) Is there a relationship between processing of emotion and motor behavior in FND? (3) Are functional neurological symptoms modulated by social context in a similar way as emotional expressions? (4) Is bodily awareness reduced in FND?

In the review, we focus on the two most prevalent subtypes of FND: psychogenic non-epileptic seizures (PNES) and functional movement disorders (FMD). Even though there is a debate on whether these two classes of symptoms constitute separate disorders (they differ in a few respects such as age at symptom onset and comorbidities) the functional neurological symptoms commonly co-occur and unifying pathophysiology is therefore likely (10).

We aim to bring together biomedical and psychosocial perspectives on FND. While the biomedical perspective highlights abnormalities in a person's psychological and brain functioning, the psychosocial perspective stresses that the illness occurs in individuals with a personal history within an interactional matrix including their family, health care system, and cultural context such that the symptoms may have meaning other than as signs of underlying psychological or brain pathology (11). These two broad perspectives have already been suggested in the Freudian theory of conversion hysteria, in which repression and conversion constituted intrapsychic primary gains in alleviating internal conflict, with the external motivator of illness allowing the individual to escape from difficult social tasks providing secondary gains (12). Even though recent cognitive and brain-based models largely divert from this mechanistic theory, they continue to describe conversion symptoms as a result of intrapsychic or neural process without addressing their possible meanings or social functions. We aim to bridge these two accounts by showing that FND may have a social signaling function rooted in individual pathophysiology.

## Emotion and interoception

We use recently developed Bayesian predictive coding framework applied to emotion and interoception (13–15) to integrate and understand the research findings presented in the review. A predictive account of interoception provides neurobiologically sound theory that integrates interoceptive, motor and social aspects of emotion. This approach is highly relevant for somatic disorders with hypothesized emotional etiological factors because it places emotional and bodily information within one conception thereby escaping mind-body dualism. Moreover, this framework makes it possible to show how social factors, such as secondary gains, may influence the processing of information about one's body. Predictive coding perspective has been already applied to sensorimotor aspects of FND (16) and to medically unexplained symptoms generally (17). Van den Bergh (17) describe a comprehensive model of symptom perception in medically unexplained symptoms with use of interoceptive predictive coding paradigm. However, none of the Bayesian models focus on interpersonal and social contexts that may shape the intensity of the functional neurological symptoms.

The predictive coding model of brain function is a powerful neurobiological framework postulating that the brain uses its generative model of the world to make predictions about causes of sensory data (18). The predictions are constructed from previous experiences that together constitute brain's internal models of the world. The difference between prediction (also named prior belief or expectation) and sensory data constitutes a prediction error that is used by the brain to refine predictions. By minimizing mismatches between expectations and experience, the brain tries to maximize evidence for its models of the world. Perceptual inference describes a process in which prediction error is minimized by changing the expectation. Alternatively, the brain can change the sampling of sensory data (e.g., change of perspective by head movement) to make them fit the prediction. This process is described as an active inference in a predictive coding framework (19). Whether prediction or sensory sampling is modified depends on the relative precision of the sensory data. If the sensory information is precise, the prediction is likely to change. On the other hand, if sensory input is noisy relative to precise prediction, the brain uses action to minimize prediction error.

The predictive coding framework applied to interoception puts the homeostatic regulation and sensory consequences of the regulation, i.e. interoception, at the core of the mind and brain architecture (15). In this theory, the brain is assumed to regulate homeostasis by issuing predictions about future physiological demands, e.g., the brain predicts oxygen expenditure due to movement, and fulfills these predictions by bodily adjustments, e.g. by increasing the heart rate (15). Successful energy regulation is not possible without building a proper interoceptive model of the body that generates interoceptive predictions. Agranular visceromotor cortices, including cingulate cortex, and posterior parts of the ventromedial and orbitofrontal cortex, are hypothesized to estimate predicted body requirements based on past experience (13). Interoceptive signals, such as cardiac and respiratory signals, glucose levels, and temperature, are represented in the posterior and mid insula, which serve as the primary interoceptive cortex. The anterior insular cortex is assumed to be a central region in interoceptive pathways that both detect and cause changes of the physiological condition; is also implicated in self-awareness and the salience of exteroceptive information based on personal significance (20, 21).

In humans, social interaction serves in the regulation of homeostasis (22). Because of the late maturation of the human motor system, the homeostatic regulation of infants is highly dependent on the infant's ability to signal needs and the caregiver's ability to perceive these signals and react accordingly. In other words, in infants, the regulation of homeostasis is partially “outsourced” to a caregiver and is therefore an inherently interactional process. An infant's emotional expressions, linked to its internal state (hunger, irritation, anger, etc.) elicit behavioral responses in others, and the detection of these emotional expressions by a caregiver serves as a validation of the internal state and facilitates the proper development of interoception in an infant. Interoception develops early in childhood (23) and its development is influenced by environmental factors (24); notably, childhood abuse has been shown to alter nodes within the interoceptive network (25). Altered interoceptive processing has been associated with alexithymia (26), emotional regulation (27) and development of somatic symptoms (28), all of which are implied in FND.

## Methods

### Search strategy and selection criteria

We searched the databases PubMed and Web of Knowledge from 2007 to June 2018. Search terms were (“conversion disorder” OR “functional neurological disorder” OR “psychogenic”) AND (emotion^*^ OR affect^*^). Reference lists of relevant articles were also searched. The results were assessed for inclusion using the publication title and abstract. Studies were included if they conformed to the following inclusion and exclusion criteria. Studies were included if (i) they reported on patients with functional neurological disorder described as functional, non-organic, psychogenic, hysterical, or conversion disorder; (ii) they reported data comparing cases with at least one control group; (iii) we included studies in adult as well as pediatric populations; (iv) we focused our review on experimental studies so we excluded studies that used only self-reports with exception of studies on life events where self-reporting cannot be avoided. The exclusion of self-reports is motivated by the assumption that self-reports targeting emotional processing demand certain capacity for introspection which may be diminished in the studied population of FND patients; (v) neuroimaging studies were only included if they also yielded behavioral or physiological data.

### Organization of studies

The search yielded 622 results. We selected 34 articles which met the above inclusion criteria. A summary of selected studies is presented in Table 1. The studies were categorized with respect to the four questions formulated in the introduction and their findings are summarized in the following sections.

**Table 1:** Processing of emotion in FND: a summary of studies.

**Reference**	**Symptoms**	**Illness duration**	**FND (*n*)**	**Control (*n*)**	**Stimuli**	**Task**	**Outcome measure**
(29)	Motor FND	2 years <	12	14 healthy	Sad and fearful Ekman faces	Incidental affective task	Reaction times, BOLD
(30)	Motor FND	13.5 months	12	13 healthy	Personal narratives	Event recall	Reaction times, BOLD
(31)	Motor FND	31 months	10	10 healthy	Pleasant/unpleasant IAPS pictures	Emotional-force control task	Grip force, BOLD
(32)	Functional dystonia	3.5 years	12	12 organic dystonia, 25 healthy	Emotional faces, IAPS	Incidental affective task, oddball task	BOLD
(33)	Functional tremor	4.8 years	27	16 essential tremor, 25 healthy	Emotional faces, IAPS	Incidental affective task, oddball task	BOLD
(34)	Motor FND	Unspecified	20	20 healthy	Neutral/negative IAPS	Emotion regulation task	MEG
(35)	Functional weakness	12 months	21	21 healthy	Neutral/negative IAPS	Passive watching	MEG
(36)	Functional paresis	83 weeks	13	19 healthy	Sad or calm faces	Sensorimotor stimulation	BOLD
(37)	Acute FND	1.5 months (younger), 12 months (older)	57	57 healthy	Tones	Auditory oddball	EEG
(38)	Motor FND	Unspecified	25	20 healthy	IAPS and aversive sounds	Negative conditioning	BOLD
(39)	PNES	4 years	12	12 temporal epilepsy, 24 healthy	Happy, fearful, sad, neutral faces	Incidental affective task	BOLD
(40)	Motor FND	6.4 years	16	16 healthy	Fearful, happy and neutral faces stimuli	Incidental affective task	BOLD
(41)	Motor FND	Unspecified	11	11 healthy	N/A	Action selection task	BOLD
(42)	Motor FND	4.9 years	35	35 healthy	N/A	CTQ, HAMD, HAMA, SCID, BDI, rsfMRI	BOLD, subjective rating
(43)	Motor FND	73 months	16	15 healthy	N/A	Trier social stress task	Salivary cortisol and alpha amylase
(44)	PNES	6.5 years	19	20 healthy	Backwardly masked angry, neutral, and happy faces	Masked emotional Stroop test, Trier Social Stress Test	Color-naming latencies, HRV, cortisol
(45)	PNES	6.5 years	19	20 healthy	Angry, happy and neutral faces	n-back task, cold pressor task	Working memory performance, cortisol
(46)	PNES	6.5 years	19	17 epilepsy, 20 healthy	Angry, happy and neutral faces	Emotional stroop task	Attentional bias, cortisol
(47)	PNES	Unspecified	12	20 healthy	Angry faces	Approach and avoidance task, cold pressor task	Cortisol, action tendencies
(48)	Motor FND	1.5m (younger), 12 m (older)	57	57 healthy	Six facial expressions of emotion	Emotion-identification task	Reaction time
(49)	Motor FND	1.5m (younger), 12 m (older)	57	57 healthy	Emotional faces	Oddball task, the Go/No-Go task, and the facial emotion- perception task	HRV
(50)	Motor FND	Unspecified	25	24 healthy	Facial expressions of emotion	Emotion-identification task	Reaction time, brain volume
(51)	PNES	60 m	39	42 healthy	IAPS pictures	Valence/arousal rating	SCR, SCL, subjective rating
(51)	PNES	54 m	38	43 healthy	Angry, happy, neutral faces	Pictorial emotional Stroop test	Color-naming errors, RT, attentional bias score
(52)	PNES	Unspecified	18	18 PTSD-high, 18 PTSD-low	IAPS pictures	Valence/arousal rating	Subjective rating, cardiac interbeat interval (IBI) and respiratory sinus arrhythmia (RSA)
(53)	Motor FND	7 years	16	17 healthy	N/A	HB detection task	HB accuracy
(54)	Motor FND	37.44 m	12	12 healthy	neg, pos, neu IAPS pictures	Modulation of the startle eyeblink reflex	EMG, subjective rating
(55)	PNES	Unspecified	22	22 healthy	Auditory bursts	Auditory startle response	EMG
(56)	PNES, motor FND, fibromyalgia	Unspecified	41	41 organic neurological disorder	N/A	CTQ, LEC, DERS, PLC-5, HADS	Subjective rating
(57)	Motor and sensory FND	Unspecified	60	39 PTSD, 40 healthy	N/A	SDQ-20, DES, PDS, MACE, TAS-26	Subjective rating
(58)	Motor FND	6 years	64	39 focal hand dystonia, 38 healthy	N/A	CTQ, TLEQ, SRS, PBI, NEO PI-R, BAI, BDI, DES	Subjective rating
(59)	PNES	Unspecified	56	59 healthy	N/A	CTQ, DIS-Q	Subjective rating
(60)	Motor and sensory FND	Unspecified	45	45 healthy	N/A	CTQ, TLEQ, SRS, PBI, NEO PI-R, baI, BDI, DES	Subjective rating
(61)	FND	Unspecified	34 studies with 1405 FND		N/A	Meta-analysis	N/A

#### Sensitivity to distress in FND

A number of reviewed studies have provided evidence for higher autonomic sensitivity to emotional stimuli, especially threat signals in FND patients. Seignourel et al. (54) found increased startle reactions to positive and negative pictures in FND patients with no effect of depression or anxiety scores on startle modulation. Yalcin et al. (55) reported increased orienting responses in PNES patients but no difference in auditory startle reaction as compared to healthy participants. The orienting response facilitates attention to a stimulus and the response was shown to be associated with cortical processing, especialy in ventromedial prefrontal cortex and anterior cingulate cortex (62). Bakvis (44) found PNES patients to be more vigilant for social threat stimuli; this increased threat vigilance was related to self-reported trauma. Bakvis (46) also demonstrated a positive correlation between baseline cortisol levels and attentional bias scores for threat stimuli that was specific only to PNES patients and was absent in epilepsy patients and healthy subjects. PNES patients also showed increased avoidance tendencies to social threat cues (47) but other study showed that avoidance learning is impaired in heterogenous FND group (38). In a pediatric FND patients, Kozlowska et al. (37) found increased cortical arousal during an auditory oddball task and the same research group also demonstrated high autonomic arousal at baseline and in response to emotional faces in children with FND (49).

High sensitivity to threat signals and motor mobilization in FND have led several authors to hypothesize that functional neurological symptoms represent forms of human defensive behavior in response to threat (31, 63). However, several studies showed that increased autonomic arousal found in FND is not specific to threat signals (fear and anger faces used mostly in research studies); it has also been reported in perceptions of positive emotional displays (40, 45, 51, 54) suggesting a generalized state of hyperarousal in FND.

With regard to predisposing factors associated with FND, theoretical models and research have traditionally focused on traumatic events such as sexual or physical abuse (64). Dramatic presentations such as nonepileptic seizures or functional gait disorders may motivate a search for equally traumatic triggers. However, the role of trauma in the etiology of FND remains controversial. The presence of traumatic events in the patient's personal history was left out of the main DSM criteria for FND because it is difficult to prove a causal link between life events and symptom onset. Furthermore, some studies question the relevance of traumatic events in the etiology of the disorder because not all patients report a history of traumatic or adverse life events (57, 58, 64).

On the other hand, not all adverse events reported by patients may conform to the definition of trauma as described in the DSM and in the definitions derived from the DSM used in research studies. The DSM-5 definition of trauma requires “actual or threatened death, serious injury, or sexual violence” (9). Stressful events not involving an immediate threat to life or physical injury, such as psychosocial stressors (divorce, job loss, illness in the family) are not considered trauma by this definition (65). However, more subtle and chronic traumatization such as emotional abuse and the presence of physical or mental illness in family have been shown to have a great impact on the subsequent levels of an individual's psychological functioning (66).

Several reviewed studies suggest relevance of adverse interpersonal relating, such as emotional abuse and neglect in FND. Emotional abuse includes verbal abuse, constant criticism, intimidation, or manipulation over a prolonged period of time (67). Emotional neglect can be considered a subtype of emotional abuse and represents a parent's failure to respond enough to a child's emotional needs and failure to provide touch, affection, nurturance, and attention (68). One recent study showed that the frequency of emotional abuse, but not physical or sexual abuse, significantly differed between FND and healthy controls (60). Karatzias et al. (56) found that childhood physical neglect was significantly associated with FND. Another research group found emotional neglect had a stronger association with the development of FND than physical or sexual abuse (61). Specifically in PNES, Ozcetin et al. (59) found emotional abuse and emotional neglect to be significantly more frequent than in healthy controls. Kruger and Fletcher (69) showed that childhood emotional neglect by the biological parents and later emotional abuse by intimate partners predict the development of dissociative disorders. Importantly, emotional abuse has been associated with insular volume (70) and abnormal connectivity between the right TPJ and left insula (42) in FND patients. The right TPJ has been associated with body-related attentional processes and social cognition (71), and the insular cortex has been repeatedly implicated in processing interoceptive information and emotional awareness (15). Negative childhood experiences modulate the prefrontal-insular-motor cortical network (72) and cumulative adversity alters connectivity and the gray matter volume of nodes within the interoceptive network, even in healthy adults (25, 73). Even if subtle forms of traumatization such as emotional abuse are prevalent in the general population and may not be specific to FND, these experiences may induce significant biological changes and, thus, influence the physiological response to stress in adult life.

#### Emotion-motion interactions in FND

We summarize the body of neuroimaging evidence addressing motor activation during the processing of emotional information in FND patients and further provide an explanatory framework for the reviewed evidence. Several recent studies found distinct patterns of connectivity between limbic regions and motor areas in FND. In an fMRI study, Voon et al. (40) found patients with functional motor disorders having higher functional connectivity between the amygdala and supplementary motor area (SMA) during processing of both positive and negative emotional stimuli. Similarly, in a study by Aybek et al. (29), FND patients showed higher amygdalar activity in response to fearful faces, accompanied by increased activity in the SMA and periaqueductal gray matter, than healthy controls did. In a different paradigm Aybek et al. (30) also found increased activity of the SMA and temporo-parietal junction (TPJ) during recall of emotional memory in FND patients as well as increased functional connectivity between the SMA and amygdala. Specifically in PNES patients, Szaflarski et al. (39) found altered facial emotion processing, as compared with epilepsy patients, which was associated with abnormal motor (putamen) and limbic (parahippocampal gyrus) activations. Abnormal activation in motor areas during emotional tasks was demonstrated also in functional dystonia patients who showed decreased activity in motor cortex bilaterally when compared with patients with primary organic dystonia (32).

Two recent neuroimaging studies investigated the relationship between emotional processing and motor activity directly by manipulating both motor inputs and the emotional valence of the presented stimuli. The finding of increased SMA-amygdala connectivity was replicated by Hassa et al. (36) in patients with functional paresis during passive movement of the paretic hand while patients were watching negative emotional pictures. Blakemore et al. (31) found that FND patients maintained higher force during hand-grip while exposed to negative (but not positive) emotional stimuli relative to healthy participants. The higher force production in patients was associated with activation in the cerebellar vermis, hippocampus, and posterior cingulate cortex; healthy participants engaged the medial prefrontal cortex and inferior frontal cortex, areas associated with motor control.

In a magnetoencephalography (MEG) study, Fiess et al. (34) found that motor FND patients activated areas corresponding to the sensorimotor cortex during emotion regulation but lacked the frontocortical activity seen in healthy controls. During the rapid visual presentation of emotionally salient stimuli with the use of MEG, Fiess et al. (35) found that the automatic detection of emotional salience is unchanged in patients with FND, but involves an emotion-processing network spanning the posterior and sensorimotor areas. Interestingly, a more pronounced involvement of the sensorimotor areas during emotional stimulation was found in participants with high alexithymia scores, i.e., in participants with reduced emotional awareness.

Even during action generated without emotional stimulus, Voon et al. (41) reported lower SMA and higher amygdala, anterior insula, and posterior cingulate activity in conversion motor patients relative to controls in a purely motor task. Bryant and Das (74) reported a case study of functional (conversion) mutism with abnormal connectivity between the amygdala and motor speech center that diminished after the successful treatment of the patient. In concordance with task-based studies, van der Kruijs et al. (75) reported increased functional connectivity between regions involved in emotion and self-perception (insula) and motor preparation (precentral sulcus) in a resting-state fMRI study with PNES patients. Recently, Kozlowska (50) found greater SMA gray matter volume in children with FND associated with faster reaction times in an emotion-recognition task.

The reviewed articles revealed task-based co-activations between limbic structures and motor areas, especially the SMA, in FND. The SMA is activated by a range of tasks that require motor planning (76); it has also been implicated in the processing of emotional information. Oliveri et al. (77) stimulated the SMA with transcranial magnetic stimulation (TMS) during emotional and non-emotional visually cued movements and found increased motor readiness specifically in emotional contexts after SMA stimulation. The involvement of the SMA in emotional processing was replicated by Rodigari and Oliveri (78), who found that rTMS trains over the SMA increased skin conductance and perceived valence of emotionally negative visual stimuli. The authors concluded that the SMA could interface the limbic and motor systems in the transformation of emotional experiences into motor actions (77). Specific SMA connectivity changes have been also shown after listening to dismissive attachment narratives (79) and in affective empathy research (80).

Beyond the known subcortical-motor pathways that mediate automatic and stereotypical motor behaviors in animals and humans in reaction to threat (81, 82), there are several studies documenting limbic system connections to cortical motor-related areas that may mediate complex emotional behaviors. Specific amygdala-motor interactions have been implicated in generating facial expressions in monkeys (83). Similar to animal neuronal tracing studies (84, 85), a few studies suggest the existence of an amygdala-motor pathway in humans. Grezes et al. (86) found direct tracts between the amygdala and cortical motor-related areas including the SMA using diffusion tensor imaging on a large data sample from the Human Connectome Project. Recently, Toschi et al. (87) reported the existence of a distinct amygdala-motor functional network at rest in a large sample of healthy subjects. In humans, the amygdala and motor-related areas have consistently shown coactivation and functional connectivity during the perception of threatening emotional expressions (88). But connectivity between the amygdala and premotor areas may have a more general meaning. In a recent study, Diano et al. (89) examined patterns of activations during the observation of different classes of emotional expressions and found increased functional connectivity between the amygdala and premotor cortices across all observed classes of emotions, suggesting that observing emotional stimuli increases motor excitability and may reflect approach and avoidance preparation, motor mimicry, or emotional contagion.

Although the motor system has been thus far studied mostly apart from the limbic system, and there is a lack of evidence for a specific meaning of limbic-SMA interactions, a few reviewed studies suggested a possible role of the SMA in transforming emotional experience into motor actions. In their FND research, Voon et al. (41) proposed that in an arousing context, abnormal SMA-amygdala connectivity “may facilitate the expression of salient previously learned and mapped conversion motor representations” (s. 2402). The question remains of the context in which such a behavior is learned and motivated. Interestingly, increased resting-state amygdala-SMA connectivity has been reported in adolescents with nonsuicidal self-harm tendencies (90) which are viewed as habitual behaviors influenced by negative affect. These forms of behavior have been shown to be greatly influenced by social conditioning, i.e. by attention or by avoiding stressful social situations (91). The selection of adaptive behavioral responses in specific social contexts, such as signaling approach or avoidance may be relevant for FND.

#### Social modulation of functional neurological symptoms

Two inherent features of emotional expressions are that they influence the behavior of others and are also influenced by social context (92). For example, the intensity of a smile or a pained expression is dependent on social attention and the perceived approval of others (93, 94). If functional neurological symptoms are shaped by social context, it may be concluded that they are similar to expressive behaviors such as emotions. However, in contrast to social modulation of pain, the experimental research of social modulation of FNS is almost non-existent in the reviewed literature and the provided evidence for social shaping of functional neurological symptoms is only indirect.

Serious “escape events” preceding FND onset (95) and increased motor activations in FND when reading “escape event” scripts reported by Aybek et al. (30) provide important evidence that the development of functional neurological symptoms may be sensitive to social context. An escape event is a situation in which signaling symptoms influence social context in favorable way. For example, a patient developing functional paralysis can prevent his partner from ending their relationship. Aybek et al. (30) showed that exposure to an escape event description is associated with distinct activations in the right SMA and the right TPJ; brain regions implicated in motor planning and self-consciousness. Similarly, Bryant and Das (74) report that functional (conversion) mutism together with abnormal amygdala-motor connectivity diminished after treatment that targeted motivational factors; in the reported case, it was a motive to remain distant from stressful work duties.

We draw another support for the social modulation of functional neurological symptoms from placebo effect and hypnotic suggestibility research in FND. Recent evolutionary accounts of animal signaling propose that symptoms of illness may have a signaling function with the goal of shaping the behaviors of conspecifics (96). In this perspective, signaling illness can elicit social support and nurturance from others and also reduce aggression and hostility (97). Illness or injury signaling has been documented in animals (98) and as a cultural phenomenon (99). Fotopoulou and Tsakiris (22) noted that the first thing children do after scraping a knee or incurring a similar mild injury is turn to the parent and await their reaction before proceeding with their own behavioral reaction. Reaction to pain has been shown to be modulated to a great extent by expected or perceived social attention and reaction (94). Behaviors similar to FND symptoms, such as abnormalities in posture, temporally uncoordinated movements, movement stereotypes, freezing behaviors, and staring expressions have been observed in children with “disorganized” attachment whose caregivers may exhibit frightening or frightened behavior, be psychologically unavailable to the child, or themselves have unresolved traumatic experiences (100). Such salient behaviors may play a twofold role in child-parent interactions. First, they heighten the likelihood of mobilizing help and treatment even in the dismissive caregiver. Second, they may represent an exaggerated appeasement display or a feigned helpless strategy (101) that functions as a means of reducing aggression from a person upon whom the child depends.

Cultural anthropology research has repeatedly documented that people use physical symptoms to communicate distress in socially acceptable ways (11, 99). For example, *ataques de nervios* is a phenomenon similar to PNES that is common in Latin American societies; it includes fainting, trembling, or convulsions that people use to communicate distress as a way to elicit social support (102). Generally, people with lower levels of social support and low social capital report greater levels of psychosomatic symptoms (103).

The high responsiveness to placebo and nocebo interventions common in various subtypes of FND may also be evidence of the signaling function of functional neurological symptoms. A placebo is defined as a set of behaviors suggesting a clinical benefit (such as inert substance administration and sham physical treatment) or a set of behaviors provoking symptoms in the case of nocebo (104). These medical rituals may elicit or attenuate functional neurological symptoms as appropriate reactions to offered help or attention in a way similar to how social situations elicit or repress certain behaviors. Responsiveness to symptom provocation has been documented in PNES, with a seizure provoked by a saline injection or a mere verbal suggestion (105), and in functional tremor, which can be provoked by applying a tuning fork to a limb (106). Symptom decline after placebo administration has been documented in functional movement disorders (107). Ricciardi and Edwards (108) reported immediate response to botulotoxin in functional dystonia patients even though it takes botulotoxin a few days to take action.

Higher hypnotic suggestibility reported in FND and strikingly similar neural correlates in experiments matching functional symptoms with clinical analogs created by suggestion (109) are in the same line of evidence. Hypnosis can be conceptualized as a non-deceptive placebo (110)—it is a ritualized set of behaviors aiming to elicit a desired response from a hypnotized subject. A higher hypnotic suggestibility in FND patients thus indicates the propensity of patients to react in the desired direction and fulfill the expectations created by a social context.

#### Body awareness in FND

Several reviewed studies provide evidence for abnormal interoceptive awareness in FND. Ricciardi et al. (53) reported decreased cardiac interoceptive accuracy in patients with motor FND; this is assumed to reflect trait awareness of interoceptive sensations. Interestingly, the same authors also showed that lower interoceptive sensitivity predicted the tendency of patients with FND to focus on external aspects of the body (53). A marked difference in subjective and objective symptom reports in FND has also been observed; FND patients tend to over-report somatic symptoms, while clinical assessment (111) or actigraphy specifically in functional tremor (112), show low symptom frequency. Similar dissociation was also found between biological and perceived stress in FND (43). In functional tremor patients, Espay et al. (33) found increased activation in paracingulate gyrus which is associated with externally oriented cognitive style, one of the alexithymia dimensions (113). In two recent meta-analyses, Perez et al. (114) and Boeckle et al. (115) summarized consistent patterns of abnormal activations in the ACC and insula in motor FND patients, i.e., in two principal brain regions within the interoceptive network. Cingulo-insular structural alteration has been reported in female FND patients (70). Specifically, reduced left insular volume was shown to be correlated with subjective symptom severity in FND (70).

### Synthesis and discussion of the findings

The present review sought to summarize support for the hypothesis that functional neurological symptoms are emotional expressions of distress. We derived our hypothesis from the conception of conversion hysteria postulated by Freud and Breuer (116) and selected four areas of interest we now discuss further.

### Affective excitation in FND

The reviewed experimental research studies provide evidence for higher levels of affective excitation in FND postulated by Breuer and Freud (3). Such excitation seems to be generalized for various emotions and it was shown to be present on the level of cortical and autonomic arousal. Moreover, there is also growing body of evidence supporting presence of impaired child-caregiver bonds in FND such as emotional abuse or neglect. Taken together, we propose that due to adverse family environment, FND patients may fail to learn self-regulation strategies when faced with arousing stimuli. The notion that child-caregiver bonds facilitate development of the brain's major self-regulatory mechanisms has considerable empirical support (117). The ability to regulate arousal in infants has been associated with the quality of caregiving they receive (118) and children with poorer quality maternal-child relationships display poorer vagal regulation and lower heart rate acceleration (119). The impaired arousal regulation in FND thus may be partially caused by impaired close social bonds (such as emotional neglect) and not necessarily by repeated exposure to threat.

From the developmental point of view, emotional abuse and neglect, evidenced in FND by several studies, is potentially harmful also for the proper development of interoceptive brain networks. Lack of attunement between child and dismissive caregiver may cause models of internal bodily processes to be inefficient in predicting sensory inputs. Interoceptive signals then become imprecise and orient child more to external aspect of the body in the state of higher arousal. Later on, when primed by experience with illness or injury, the higher precision of exteroceptive inputs relative to interoceptive inputs may lead to symptom onset as falsely inferred cause of emotional distress.

### Processing of emotion and motor behavior in FND

The finding of abnormal limbic-motor interactions in a reaction to emotional stimuli seems to be consistent in the reviewed literature. However, there is a debate about the meaning of the limbic-motor interactions among researchers. Two main hypotheses for the finding have been suggested in the reviewed literature: (i) defense mechanism akin to freezing behavior in animals (30, 31), and (ii) previously learned motor conversion representation (Voon). We shortly discuss the proposed explanatory frameworks respectively.

Blakemore et al. (31) interpret their finding of higher grip force in reaction to negative stimuli in FND as giving evidence for similarity between animal defense mechanisms and functional neurological symptoms—similarity already postulated by Kretchmer and Nijenhuis. However, clinical evidence shows that functional neurological symptoms are oftentimes pronounced in the presence of another person in the context of receiving help and attention, e.g., in the context of medical care that is not necessarily threatening. Moreover, defensive behaviors are highly stereotyped reactions (120), on the other hand conversion motor symptoms vary greatly among patients spanning convulsions, paralysis, dystonias, gait and speech abnormalities, and other motor impairments so symptoms do not always appear to have analogs in defense behaviors. Individuals with a history of adverse life events, which is a common factor in many psychopathologies, show pronounced freeze reactions (121). Increased motor mobilization and autonomic sensitivity to emotional stimuli may therefore represent a common feature in multiple psychiatric disorders. Kozlowska (122) proposed that an impaired prefrontal cortex function due to prolonged exposure to stress may lead to impaired motor-executive functions and strengthened affect-driven motor reactions. This can be the case especially in PNES patients whose convulsions are precipitated by a higher state of arousal and followed by a parasympathetic state, suggesting a role of abnormal movement in the regulation of accumulated arousal (123). Future FND studies should include patients with anxiety, depression and other psychiatric disorders as control groups to disentangle general motor mobilization and autonomic sensitivity from limbic-motor interactions specific to FND.

Voon et al. (41) propose that functional neurological symptoms represent a pattern of movement established perhaps by a previous triggering event. This proposal is based on the observation that physical precipitating factors such as injury or illness are often present at symptom onset. Such an event may provide an explanation for bodily noise caused by chronically increased arousal (e.g., muscle tension, trembling, etc.) and gradually develop into an illness prior belief. Voon et al. (41) suggest that in the arousing situation, amygdala-SMA complex is aberrantly engaged and may facilitate expression of previously learned conversion motor representations. According to Edward et al. (16), abnormal self-directed attention may increase precision of the conversion representation and may cause movement or percepts in keeping with this prior belief. Although this theoretical account explains several clinical features of FND, the postulated association between symptoms and limbic-motor activations are only indirect because most of the reviewed studies focused either on motor or emotional variables. Moreover, to our knowledge, there is no research that would examine association between illness beliefs and limbic-motor interactions in FND.

We can only suggest that the proposed explanatory models for the FND are not mutually exclusive and further speculate that the symptoms serve as protective mechanisms patients use to cope with arousing situations (e.g., avoidance tendencies) and these mechanisms are shaped by previous experience with illness or injury. We also propose that the onset of functional neurological symptoms may be motivated by perceived or expected social reactions (e.g., avoiding unpleasant tasks, obtaining care and attention, lowering demands, more control over difficult situation etc.).

### Social modulation of functional neurological symptoms

In our review, we identified only two studies that indirectly examined social modulation of functional neurological symptoms. However, there is (mostly clinical) evidence for the influence of suggestion and placebo on the intensity of functional neurological symptoms. Functional neurological symptoms seem to be sensitive to motivational factors that a patient receive from his immediate environment. As illness or injury is embedded in social system (family, healthcare), illness behavior may be shaped by behavior of others. In childhood, abnormal behaviors similar to the symptoms of a disease may be one of the limited ways how to elicit nurturance in a psychologically absent caregiver. Bowlby (124) suggested that the attachment relationship generates internal working models of self with other through repeated iterations that come to act as templates on which further relationships are build. In the terms of predictive coding account, prior experience with others' reactions to illness influences subsequent predictions of social outcome related to illness. Anticipated or offered help provided to patients by caregivers may evoke previously learned mental models of social relating—if a patient is inclined by prior experiences to expect potential help from others only during bodily threat (during illness, disease), the patient may experience and react to body-related prediction errors differently than when others' support is available. We therefore hypothesize that the emergence of symptoms may be motivated by their predicted social outcome. In 1986, Taylor proposed that hysteria is inseparable from medical care where it gains its validity by repeated examinations and attention from medical professionals (125). Repeated medical examinations may also increase the precision of prior illness beliefs by nocebo conditioning, consequently affecting active inferential processes and ultimately facilitating the development of symptoms.

### Body awareness in FND

Several reviewed studies showed evidence for impaired interoceptive awareness in FND. Edwards et al. (16) has already postulated abnormal body-centered attention as a potential mechanism behind functional neurological symptoms. Although we are sympathetic to this Bayesian framework, the authors focus mainly on sensorimotor system in FND without addressing emergent evidence of impaired interoceptive awareness in FND. The presented research findings suggest abnormal bodily perception in FND in a way that external aspects of the body are given more weight than internal inputs. If interoceptive signals are viewed as highly ambiguous prediction errors, they are more prone to misinterpretations when integrated with more precise external inputs. The ventriloquist effect is an example of the Bayesian integration of two sensory inputs, where an auditory input is bound to a visual input, such that they appear, falsely, to co-occur spatiotemporally (126). The precision of each input is estimated and weighted relative to the other; imprecise auditory input is given less weight than a more precise visual input. Their co-location is determined accordingly, forming the illusion of a sound located in the mouth of a puppet. In an analogy to the ventriloquist effect, ambiguous interoceptive signals (visceral discomfort) are weighted less than precise tactile signals (hand sweating, dry mouth), proprioceptive sensations, and visual (body trembling) information, causing a state of anxiety to be misinterpreted as a bodily symptom instead of a complex emotion. This effect can be pronounced, especially in the context in which prior expectation is primed by preceding experience with unrelated illness or injury. The weight of exteroceptive inputs relative to interoceptive signals may also be amplified by abnormal attentional resources directed toward external aspects of the body commonly observed in FND patients (8). Functional neurological symptoms then arise as a falsely inferred cause of emotional distress resulting from the Bayesian integration of imprecise interoceptive information with relatively precise exteroceptive information. These symptoms may become new forms of emotional reactions in stressful situations and also subjects of reinforcement by actual social contexts or predicted social outcomes as described above.

## Conclusion and future directions

In the review, we showed that emotional processing is an important factor in the etiology of FND. Taken together, we conclude that limbic-motor interactions evidenced in FND may reflect learned emotional behaviors of an individual with low interoceptive (and emotional) awareness and we interpret functional neurological symptoms as forms of complex affective reactions to stress similar to emotional expressions. Future studies should focused on examining brain activations in FND patients in response to stimuli relevant for the disorder, such as attachment narratives or autobiographical information. Moreover, exploring an effect of social context on the intensity of functional neurological symptoms could provide new information about the function of the symptoms.

## Author contributions

The literature search for the study was managed by PS. The first draft of the review was written by PS and MB. TK and MS critically revised and commented on the manuscript and figures. All the authors substantially contributed to and have approved the final manuscript.

### Conflict of interest statement

The authors declare that the research was conducted in the absence of any commercial or financial relationships that could be construed as a potential conflict of interest.

## References

[1] Stone J Warlow C Sharpe M. The symptom of functional weakness: a controlled study of 107 patients. Brain (2010) 133(Pt 5):1537–51. 10.1093/brain/awq068 20395262

[2] Brown RJ Reuber M. Psychological and psychiatric aspects of psychogenic non-epileptic seizures (PNES): a systematic review. Clin Psychol Rev. (2016) 45:157–82. 10.1016/j.cpr.2016.01.003 27084446

[3] Breuer J Freud S Studies on Hysteria (1957). Oxford: Basic Books.

[4] Szasz T The Myth of Mental Illness. London:Secker and Warburg (1962).

[5] Shoenberg PJ The symptom as stigma or communication in hysteria. Int J Psychoanal Psyhother. (1975) 4:507–17. 1158609

[6] Kretschmer E Hysteria: Reflex and Instinct. London: Peter Owen (1948).

[7] Nijenhuis ERS Vanderlinden J Spinhoven P Vanderlinden J van DyckR van der HartO. Somatoform dissociative symptoms as related to animal defensive reactions to predatory imminence and injury. J Abnorm Psychol. (1998) 107:63–73. 10.1037/0021-843X.107.1.63 9505039

[8] Edwards MJ Fotopoulou A Pareés I. Neurobiology of functional (psychogenic) movement disorders. Curr Opin Neurol. (2013) 26:442–7. 10.1097/WCO.0b013e3283633953 23823467 PMC4196785

[9] American Psychiatric Association Diagnostic and Statistical Manual of Mental Disorders. Washington, DC: American Psychiatric Association (2013).

[10] Edwards MJ. Neurobiologic theories of functional neurologic disorders. Handb Clin Neurol. (2017) 139:131–7. 10.1016/B978-0-12-801772-2.00012-6 27719834

[11] Kirmayer LJ Santhanam R The anthropology of hysteria. In: Halligan PW Bass C Marshall JC editors. Contemporary Approaches to the Study of Hysteria: Clinical and Theoretical Perspectives. Oxford: Oxford University Press (2001), p. 251–70.

[12] Kanaan RAA. Freud's hysteria and its legacy. Handb Clin Neurol. (2016) 139:37–44. 10.1016/B978-0-12-801772-2.00004-7 27719857

[13] Barrett LF Simmons WK. Interoceptive predictions in the brain. Nat Rev Neurosci. (2015) 16:419–29. 10.1038/nrn3950 26016744 PMC4731102

[14] Chanes L Barrett LF. Redefining the role of limbic areas in cortical processing. Trends Cogn Sci. (2015) 20:96–106. 10.1016/j.tics.2015.11.005 26704857 PMC4780414

[15] Barrett L Quigley KS Hamilton P. An active inference theory of allostasis and interoception in depression. Philos Trans R Soc Lond B Biol Sci. (2016) 371:20160011. 10.1098/rstb.2016.0011 28080969 PMC5062100

[16] Edwards MJ Adams RA Brown H Pareés I Friston KJ. A Bayesian account of “hysteria”. Brain (2012) 135(Pt 11):3495–512. 10.1093/brain/aws129 22641838 PMC3501967

[17] Van den Bergh O Witthöft M Petersen S Brown R Symptoms and the body: taking the inferential leap. Neurosci Biobehav Rev. (2017) 74:185–203. 10.1016/j.neubiorev.2017.01.015 28108416

[18] Friston K. The free-energy principle: a unified brain theory? Nat Rev Neurosci. (2010) 11:127–38. 10.1038/nrn2787 20068583

[19] den Ouden HEM Kok P de Lange FP How prediction errors shape perception, attention, and motivation. Front Psychol. (2012) 3:548 10.3389/fpsyg.2012.00548 23248610 PMC3518876

[20] Craig ADB. How do you feel–now? The anterior insula and human awareness. Nat Rev Neurosci. (2009) 10:59–70. 10.1038/nrn2555 19096369

[21] Gu X Hof PR Friston KJ Fan J. Anterior insular cortex and emotional awareness. J Compar Neurol. (2013) 521:3371–88. 10.1002/cne.23368 23749500 PMC3999437

[22] Fotopoulou A Tsakiris M Mentalizing homeostasis: the social origins of interoceptive inference. Neuropsychoanalysis (2017) 19:3–28. 10.1080/15294145.2017.1294031

[23] Koch A Pollatos O. Cardiac sensitivity in children: sex differences and its relationship to parameters of emotional processing. Psychophysiology (2014) 51:932–41. 10.1111/psyp.12233 24810627

[24] Ambrosecchia M Ardizzi M Russo E Ditaranto F Speciale M Vinai P. Interoception and autonomic correlates during social interactions. implications for anorexia. Front Hum Neurosci. (2017) 11:219. 10.3389/fnhum.2017.00219 28567008 PMC5434670

[25] Teicher MH Anderson CM Ohashi K Polcari A. Childhood maltreatment: altered network centrality of cingulate, precuneus, temporal pole and insula. Biol Psychiatry (2014) 76:297–305. 10.1016/j.biopsych.2013.09.016 24209775 PMC4258110

[26] Brewer R Cook R Bird G. Alexithymia: a general deficit of interoception. R Soc Open Sci. (2016) 3:150664. 10.1098/rsos.150664 27853532 PMC5098957

[27] Füstös J Gramann K Herbert BM Pollatos O. On the embodiment of emotion regulation: interoceptive awareness facilitates reappraisal. Soc Cogn Affect Neurosci. (2013) 8:911–7. 10.1093/scan/nss089 22933520 PMC3831556

[28] Schaefer M Egloff B Witthöft M. Is interoceptive awareness really altered in somatoform disorders? Testing competing theories with two paradigms of heartbeat perception. J Abnorm Psychol. (2012) 121:719–24. 10.1037/a0028509 22642840

[29] Aybek S Nicholson TR O'Daly O Zelaya F Kanaan RA David AS. Emotion-motion interactions in conversion disorder: an FMRI study. PloS ONE (2015) 10:e0123273. 10.1371/journal.pone.0123273 25859660 PMC4393246

[30] Aybek S Nicholson TR Zelaya F O'Daly OG Craig TJ David AS. Neural correlates of recall of life events in conversion disorder. JAMA Psychiatry (2014) 71:52–60. 10.1001/jamapsychiatry.2013.2842 24258270

[31] Blakemore RL Sinanaj I Galli S Aybek S Vuilleumier P. Aversive stimuli exacerbate defensive motor behaviour in motor conversion disorder. Neuropsychologia (2016) 93(Pt A):229–41. 10.1016/j.neuropsychologia.2016.11.005 27842291

[32] Espay AJ Maloney T Vannest J Norris MM Eliassen JC Neefus E. Dysfunction in emotion processing underlies functional (psychogenic) dystonia. Move Disord. (2018) 33:136–45. 10.1002/mds.27217 29124784 PMC5767134

[33] Espay AJ Maloney T Vannest J Norris MM Eliassen JC Neefus E. Impaired emotion processing in functional (psychogenic) tremor: a functional magnetic resonance imaging study. NeuroImage Clin. (2018) 17:179–87. 10.1016/j.nicl.2017.10.020 29085776 PMC5655406

[34] Fiess J Rockstroh B Schmidt R Steffen A. Emotion regulation and functional neurological symptoms: does emotion processing convert into sensorimotor activity? J Psychosomat Res. (2015) 79:477–83. 10.1016/j.jpsychores.2015.10.009 26652591

[35] Fiess J Rockstroh B Schmidt R Wienbruch C Steffen A. Functional neurological symptoms modulate processing of emotionally salient stimuli. J Psychosomat Res. (2016) 91:61–7. 10.1016/j.jpsychores.2016.10.007 27894464

[36] Hassa T Sebastian A Liepert J Weiller C Schmidt R Tüscher O. Symptom-specific amygdala hyperactivity modulates motor control network in conversion disorder. NeuroImage Clin. (2017) 15:143–50. 10.1016/j.nicl.2017.04.004 28529870 PMC5429234

[37] Kozlowska K Melkonian D Spooner CJ Scher S Meares R. Cortical arousal in children and adolescents with functional neurological symptoms during the auditory oddball task. NeuroImage Clin. (2017) 13:228–36. 10.1016/j.nicl.2016.10.016 28003962 PMC5157791

[38] Morris LS To B Baek K Chang-Webb Y-C Mitchell S Strelchuk D. Disrupted avoidance learning in functional neurological disorder: implications for harm avoidance theories. NeuroImage Clin. (2017) 16:286–94. 10.1016/j.nicl.2017.08.007 28856091 PMC5562176

[39] Szaflarski JP Allendorfer JB Nenert R LaFrance WC Barkan HI DeWolfe J. Facial emotion processing in patients with seizure disorders. Epilepsy Behav. (2018) 79:193–204. 10.1016/j.yebeh.2017.12.004 29309953

[40] Voon V Brezing C Gallea C Ameli R Roelofs K LaFrance WC. Emotional stimuli and motor conversion disorder. Brain (2010) 133(Pt 5):1526–36. 10.1093/brain/awq054 20371508 PMC2859149

[41] Voon V Brezing C Gallea C Hallett M. Aberrant supplementary motor complex and limbic activity during motor preparation in motor conversion disorder. Move Disord. (2011) 26:2396–403. 10.1002/mds.23890 21935985 PMC4162742

[42] Maurer CW LaFaver K Ameli R Epstein SA Hallett M Horovitz SG. Impaired self-agency in functional movement disorders. Neurology (2016) 87:564–70. 10.1212/WNL.0000000000002940 27385746 PMC4977370

[43] Apazoglou Biological and perceived stress in motor functional neurological disorders. Psychoneuroendocrinology (2017) 85:142–50. 10.1016/j.psyneuen.2017.08.023 28863348

[44] Bakvis P Roelofs K Kuyk J Edelbroek PM Swinkels WAM Spinhoven P. Trauma, stress, and preconscious threat processing in patients with psychogenic nonepileptic seizures. Epilepsia (2009) 50:1001–11. 10.1111/j.1528-1167.2008.01862.x 19170739

[45] Bakvis P Spinhoven P Putman P Zitman FG Roelofs K. The effect of stress induction on working memory in patients with psychogenic nonepileptic seizures. Epilepsy Behav. (2010) 19:448–54. 10.1016/j.yebeh.2010.08.026 20943444

[46] Bakvis P Spinhoven P Roelofs K. Basal cortisol is positively correlated to threat vigilance in patients with psychogenic nonepileptic seizures. Epilepsy Behav. (2009) 16:558–60. 10.1016/j.yebeh.2009.09.006 19818692

[47] Bakvis P Spinhoven P Zitman FG Roelofs K. Automatic avoidance tendencies in patients with Psychogenic Non Epileptic Seizures. Seizure (2011) 20:628–34. 10.1016/j.seizure.2011.06.006 21752672

[48] Kozlowska K Brown KJ Palmer DM Williams LM. Specific biases for identifying facial expression of emotion in children and adolescents with conversion disorders. Psychosom Med. (2013) 75:272–80. 10.1097/PSY.0b013e318286be43 23440229

[49] Kozlowska K Palmer DM Brown KJ McLean L Scher S Gevirtz R. Reduction of autonomic regulation in children and adolescents with conversion disorders. Psychosomat Med. (2015) 77:356–70. 10.1097/PSY.0000000000000184 25954919

[50] Kozlowska K Griffiths KR Foster SL Linton J Williams LM Korgaonkar MS. Grey matter abnormalities in children and adolescents with functional neurological symptom disorder. Neuroimage Clin. (2017) 15:306–14. 10.1016/j.nicl.2017.04.028 28560155 PMC5440356

[51] Pick S Mellers JDC Goldstein LH. Autonomic and subjective responsivity to emotional images in people with dissociative seizures. J Neuropsychol. (2018) 12:341–55. 10.1111/jnp.12144 29285879 PMC6001553

[52] Roberts NA Burleson MH Weber DJ Larson A Sergeant K Devine MJ. Emotion in psychogenic nonepileptic seizures: responses to affective pictures. Epilepsy Behav. (2012) 24:107–15. 10.1016/j.yebeh.2012.03.018 22520585

[53] Ricciardi L Demartini B Crucianelli L Krahé C Edwards MJ Fotopoulou A. Interoceptive awareness in patients with functional neurological symptoms. Biol Psychol. (2016) 113:68–74. 10.1016/j.biopsycho.2015.10.009 26528552

[54] Seignourel PJ Miller K Kellison I Rodriguez R Fernandez HH Bauer RM. Abnormal affective startle modulation in individuals with psychogenic [corrected] movement disorder. Move Disord. (2007) 22:1265–71. 10.1002/mds.21451 17486611

[55] Yalçin M Tellioglu E Gündüz A Özmen M Yeni N Özkara Ç. Orienting reaction may help recognition of patients with psychogenic nonepileptic seizures. Neurophysiol Clin Clin Neurophysiol. (2017) 47:231–7. 10.1016/j.neucli.2017.02.005 28314521

[56] Karatzias T Howard R Power K Socherel F Heath C Livingstone A. Organic vs. functional neurological disorders: the role of childhood psychological trauma. Child Abuse Negl. (2017) 63:1–6. 10.1016/j.chiabu.2016.11.011 27886517

[57] Kienle J Rockstroh B Bohus M Fiess J Huffziger S Steffen-Klatt A. Somatoform dissociation and posttraumatic stress syndrome - two sides of the same medal? A comparison of symptom profiles, trauma history and altered affect regulation between patients with functional neurological symptoms and patients with PTSD. BMC Psychiatry (2017) 17:248. 10.1186/s12888-017-1414-z 28693577 PMC5504809

[58] Kranick S Ekanayake V Martinez V Ameli R Hallett M Voon V. Psychopathology and psychogenic movement disorders. Move Disord. (2011) 26:1844–50. 10.1002/mds.23830 21714007 PMC4049464

[59] Ozcetin A Belli H Ertem U Bahcebasi T Ataoglu A Canan F. Childhood trauma and dissociation in women with pseudoseizure-type conversion disorder. Nordic J Psychiatry (2009) 63:462–8. 10.3109/08039480903029728 19544219

[60] Steffen A Fiess J Schmidt R Rockstroh B “That pulled the rug out from under my feet!” - adverse experiences and altered emotion processing in patients with functional neurological symptoms compared to healthy comparison subjects. BMC Psychiatry (2015) 15:133 10.1186/s12888-015-0514-x 26103961 PMC4477601

[61] Ludwig L Pasman JA Nicholson T Aybek S David AS Tuck S. Stressful life events and maltreatment in conversion (functional neurological) disorder: systematic review and meta-analysis of case-control studies. Lancet Psychiatry (2018) 5:307–20. 10.1016/S2215-0366(18)30051-8 29526521

[62] Williams LM Brammer MJ Skerrett D Lagopolous J Rennie C Kozek K. The neural correlates of orienting: an integration of fMRI and skin conductance orienting. Neuroreport (2000) 11:3011–5. 10.1097/00001756-200009110-00037 11006985

[63] Vuilleumier P. Hysterical conversion and brain function. Prog Brain Res. (2005) 150:309–29. 10.1016/S0079-6123(05)50023-2 16186033

[64] Roelofs K Pasman J. Stress, childhood trauma, and cognitive functions in functional neurologic disorders. Handb Clin Neurol. (2016) 139:139–55. 10.1016/B978-0-12-801772-2.00013-8 27719835

[65] Pai A Suris AM North CS. Posttraumatic stress disorder in the DSM-5: controversy, change, and conceptual considerations. Behav Sci. (2017) 7:7. 10.3390/bs7010007 28208816 PMC5371751

[66] Dvir Y Ford JD Hill M Frazier JA. Childhood maltreatment, emotional dysregulation, and psychiatric comorbidities. Harvard Rev Psychiatry (2014) 22:149–61. 10.1097/HRP.0000000000000014 24704784 PMC4091823

[67] Glaser D Emotional abuse and neglect (psychological maltreatment): a conceptual framework. Child Abuse Negl. (2002) 26:697–714. 10.1016/S0145-213(02)00342-3 12201163

[68] Naughton AM Maguire SA Mann MK Lumb RC Tempest V Gracias S. Emotional, behavioral, and developmental features indicative of neglect or emotional abuse in preschool children. JAMA Pediatr. (2013) 167:769. 10.1001/jamapediatrics.2013.192 23754198

[69] Krüger C Fletcher L. Predicting a dissociative disorder from type of childhood maltreatment and abuser-abused relational tie. J Trauma Dissociation (2017) 18:356–72. 10.1080/15299732.2017.1295420 28318411

[70] Perez DL Matin N Barsky A Costumero-Ramos V Makaretz SJ Young SS. Cingulo-insular structural alterations associated with psychogenic symptoms, childhood abuse and PTSD in functional neurological disorders. J Neurol Neurosurg Psychiatry (2017) 88:491–7. 10.1136/jnnp-2016-314998 28416565 PMC5497745

[71] Krall SC Rottschy C Oberwelland E Bzdok D Fox PT Eickhoff SB. The role of the right temporoparietal junction in attention and social interaction as revealed by ALE meta-analysis. Brain Struct Funct. (2015) 220:587–604. 10.1007/s00429-014-0803-z 24915964 PMC4791048

[72] Duncan NW Hayes DJ Wiebking C Tiret B Pietruska K Chen DQ. Negative childhood experiences alter a prefrontal-insular-motor cortical network in healthy adults: a preliminary multimodal rsfMRI-fMRI-MRS-dMRI study. Hum Brain Mapp. (2015) 36:4622–37. 10.1002/hbm.22941 26287448 PMC4827445

[73] Ansell EB Rando K Tuit K Guarnaccia J Sinha R. Cumulative adversity and smaller gray matter volume in medial prefrontal, anterior cingulate, and insula regions. Biol Psychiatry (2012) 72:57–64. 10.1016/j.biopsych.2011.11.022 22218286 PMC3391585

[74] Bryant RA Das P. The neural circuitry of conversion disorder and its recovery. J Abnor Psychol. (2012) 121:289–96. 10.1037/a0025076 21859163

[75] van der Kruijs SJM Bodde NMG Vaessen MJ Lazeron RHC Vonck K Boon P Functional connectivity of dissociation in patients with psychogenic non-epileptic seizures. J Neurol Neurosurg Psychiatry (2012) 83:239–47. 10.1136/jnnp-2011-300776 22056967

[76] Nachev P Kennard C Husain M. Functional role of the supplementary and pre-supplementary motor areas. Nat Rev Neurosci. (2008) 9:856–69. 10.1038/nrn2478 18843271

[77] Oliveri M Babiloni C Filippi MM Caltagirone C Babiloni F Cicinelli P. Influence of the supplementary motor area on primary motor cortex excitability during movements triggered by neutral or emotionally unpleasant visual cues. Exp Brain Res. (2003) 149:214–21. 10.1007/s00221-002-1346-8 12610690

[78] Rodigari A Oliveri M. Disrupting SMA activity modulates explicit and implicit emotional responses: an rTMS study. Neurosci Lett. (2014) 579:30–4. 10.1016/j.neulet.2014.07.012 25038415

[79] Borchardt V Krause AL Li M van Tol MJ Demenescu LR Buchheim A. Dynamic disconnection of the supplementary motor area after processing of dismissive biographic narratives. Brain Behav. (2015) 5:e00377. 10.1002/brb3.377 26516612 PMC4614061

[80] Fan Y Duncan NW de Greck M Northoff G. Is there a core neural network in empathy? An fMRI based quantitative meta-analysis. Neurosci Biobehav Rev. (2011) 35:903–11. 10.1016/j.neubiorev.2010.10.009 20974173

[81] Grèzes J Dezecache G. How do shared-representations and emotional processes cooperate in response to social threat signals? Neuropsychologia (2014) 55:105–14. 10.1016/j.neuropsychologia.2013.09.019 24080262

[82] Holstege G Chapter 14 Descending motor pathways and the spinal motor system: limbic and non-limbic components. Prog Brain Res. (1991) 87:307–421. 10.1016/S0079-6123(08)63057-5 1678191

[83] Gothard KM. The amygdalo-motor pathways and the control of facial expressions. Front Neurosci. (2014) 8:43. 10.3389/fnins.2014.00043 24678289 PMC3958699

[84] Ghashghaei HT Hilgetag CC Barbas H. Sequence of information processing for emotions based on the anatomic dialogue between prefrontal cortex and amygdala. NeuroImage (2007) 34:905–23. 10.1016/j.neuroimage.2006.09.046 17126037 PMC2045074

[85] Morecraft RJ McNeal DW Stilwell-Morecraft KS Gedney M Ge J Schroeder CM. Amygdala interconnections with the cingulate motor cortex in the rhesus monkey. J Compar Neurol. (2007) 500:134–65. 10.1002/cne.21165 17099887

[86] Grèzes J Valabrègue R Gholipour B Chevallier C. A direct amygdala-motor pathway for emotional displays to influence action: a diffusion tensor imaging study. Hum Brain Mapp. (2014) 35:5974–83. 10.1002/hbm.22598 25053375 PMC6869045

[87] Toschi N Duggento A Passamonti L. Functional connectivity in amygdalar-sensory/(pre)motor networks at rest: new evidence from the human connectome project. Eur J Neurosci. (2017) 45:1224–9. 10.1111/ejn.13544 28231395

[88] Grèzes J Dezecache G Eskenazi T Limbic to motor interactions during social perception. In: Toga AW editor. Brain Mapping. Cambridge, MA: Elsevier (2015). p. 1027–30.

[89] Diano M Tamietto M Celeghin A Weiskrantz L Tatu M-K Bagnis A. Dynamic changes in amygdala psychophysiological connectivity reveal distinct neural networks for facial expressions of basic emotions. Sci Rep. (2017) 7:45260. 10.1038/srep45260 28345642 PMC5366904

[90] Westlund Schreiner M Klimes-Dougan B Mueller BA Eberly LE Reigstad KM Carstedt PA Multi-modal neuroimaging of adolescents with non-suicidal self-injury: Amygdala functional connectivity. J Affect Disord. (2017) 221:47–55. 10.1016/j.jad.2017.06.004 28628767 PMC5555154

[91] Nock MK Prinstein MJ. A functional approach to the assessment of self-mutilative behavior. J Consult Clin Psychol. (2004) 72:885–90. 10.1037/0022-006X.72.5.885 15482046

[92] Buck R Losow JI Murphy MM Costanzo P. Social facilitation and inhibition of emotional expression and communication. J Pers Soc Psychol. (1992) 63:962–8. 10.1037/0022-3514.63.6.962 1460562

[93] Fernandez-Dols JM Ruiz-Belda M-A Mandler G Spontaneous facial behavior during intense emotional episodes: artistic truth and optical truth. In: Russell JA Fernandez-Dols JM editors. The Psychology of Facial Expression. Cambridge: Cambridge University Press (1997). p. 255–74.

[94] Krahé C Springer A Weinman JA Fotopoulou A. The social modulation of pain: others as predictive signals of salience - a systematic review. Front Hum Neurosci. (2013) 7:386. 10.3389/fnhum.2013.00386 23888136 PMC3719078

[95] Nicholson TR Aybek S Craig T Harris T Wojcik W David AS. Life events and escape in conversion disorder. Psychol Med. (2016) 46:2617–26. 10.1017/S0033291716000714 27377290 PMC4988265

[96] Steinkopf L The signaling theory of symptoms: an evolutionary explanation of the placebo effect. Evol Psychol. (2015) 13:1474704915600559 10.1177/1474704915600559 PMC10480909 37924177

[97] Tiokhin L. Do symptoms of illness serve signaling functions? (Hint: Yes). Q Rev Biol. (2016) 91:177–95. 10.1086/686811 27405223

[98] Burghardt GM Cognitive ethology and critical anthropomorphism: a snake with two heads and hognose snakes that play dead. In: Ristau CA editor. Comparative Cognition and Neuroscience. Cognitive Ethology: The Minds of Other Animals: Essays in Honor of Donald, R. Griffin. Hillsdale, NJ: Lawrence Erlbaum Associates (1991). p. 53–90.

[99] Wenegrat B Theater of Disorder : Patients, Doctors, and the Construction of Illness. Oxford University Press (2001). Available online at: https://global.oup.com/academic/product/theater-of-disorder-9780195140873?cc=cz&lang=en&

[100] Crittenden PM. Attachment and risk for psychopathology. J Dev Behav Pediatr. (1995) 16(Supp. 3):S12–6. 10.1097/00004703-199506001-00004 7560126

[101] Kozlowska K. The developmental origins of conversion disorders. Clin Child Psychol Psychiatry (2007) 12:487–510. 10.1177/1359104507080977 18095533

[102] Lewis-Fernández R Gorritz M Raggio GA Peláez C Chen H Guarnaccia PJ. Association of trauma-related disorders and dissociation with four idioms of distress among Latino psychiatric outpatients. Culture Med Psychiatry (2010) 34:219–43. 10.1007/s11013-010-9177-8 20414799 PMC4339099

[103] Åslund C Starrin B Nilsson KW. Social capital in relation to depression, musculoskeletal pain, and psychosomatic symptoms: a cross-sectional study of a large population-based cohort of Swedish adolescents. BMC Public Health (2010) 10:715. 10.1186/1471-2458-10-715 21092130 PMC3091587

[104] Benedetti F. Placebo and the new physiology of the doctor-patient relationship. Physiol Rev. (2013) 93:1207–46. 10.1152/physrev.00043.2012 23899563 PMC3962549

[105] Goyal G Kalita J Misra UK. Utility of different seizure induction protocols in psychogenic nonepileptic seizures. Epilepsy Res. (2014) 108:1120–7. 10.1016/j.eplepsyres.2014.02.015 24802296

[106] Thenganatt MA Jankovic J. Psychogenic tremor: a video guide to its distinguishing features. Tremor Other Hyperkinet Move (2014) 4:253. 10.7916/D8FJ2F0Q 25243097 PMC4161970

[107] Peckham EL Hallett M. Psychogenic movement disorders. Neurol Clin (2009) 27:801–19, vii. 10.1016/j.ncl.2009.04.008 19555832 PMC4749352

[108] Ricciardi L Edwards MJ. Treatment of functional (psychogenic) movement disorders. Neurotherapeutics (2014) 11:201–7. 10.1007/s13311-013-0246-x 24356785 PMC3899472

[109] Deeley Q Hypnosis as a model of functional neurologic disorders. Handb Clin Neurol. (2016) 139:95–103. 10.1016/B978-0-12-801772-2.00009-6 27719881

[110] Terhune DB Cleeremans A Raz A Lynn SJ. Hypnosis and top-down regulation of consciousness. Neurosci Biobehav Rev. (2017) 81(Pt A):59–74. 10.1016/j.neubiorev.2017.02.002 28174078

[111] Ricciardi L Demartini B Morgante F Parees I Nielsen G Edwards MJ. Symptom severity in patients with functional motor symptoms: patient's perception and doctor's clinical assessment. Parkinsonism Relat Disord. (2015) 21:529–32. 10.1016/j.parkreldis.2015.02.022 25772324

[112] Pareés I Saifee TA Kassavetis P Kojovic M Rubio-Agusti I Rothwell JC. Believing is perceiving: mismatch between self-report and actigraphy in psychogenic tremor. Brain (2012) 135:117–23. 10.1093/brain/awr292 22075068

[113] Lemche E Brammer MJ David AS Surguladze SA Phillips ML Sierra M. Interoceptive–reflective regions differentiate alexithymia traits in depersonalization disorder. Psychiatry Res. (2013) 214:66–72. 10.1016/j.pscychresns.2013.05.006 23932225 PMC4024664

[114] Perez DL Dworetzky BA Dickerson BC Leung L Cohn R Baslet G. An integrative neurocircuit perspective on psychogenic nonepileptic seizures and functional movement disorders : neural functional unawareness. Clin EEG Neurosci. (2014) 46:4–15. 10.1177/1550059414555905 25432161 PMC4363170

[115] Boeckle M Liegl G Jank R Pieh C. Neural correlates of conversion disorder: overview and meta-analysis of neuroimaging studies on motor conversion disorder. BMC Psychiatry (2017). 16:195. 10.1186/s12888-016-0890-x 27283002 PMC4901519

[116] Freud S Breuer J Studies on Hysteria (Basic Books Classics). New York, NY: Hogarth Press (1895/1955).

[117] Newman L Sivaratnam C Komiti A Attachment and early brain development – neuroprotective interventions in infant–caregiver therapy. Transl Dev Psychiatry (2015) 3:28647 10.3402/tdp.v3.28647

[118] McLaughlin KA Sheridan MA Tibu F Fox NA Zeanah CH Nelson CA. Causal effects of the early caregiving environment on development of stress response systems in children. Proc Natl Acad Sci USA. (2015) 112:5637–42. 10.1073/pnas.1423363112 25902515 PMC4426436

[119] Calkins SD Graziano PA Berdan LE Keane SP Degnan KA. Predicting cardiac vagal regulation in early childhood from maternal-child relationship quality during toddlerhood. Dev Psychobiol. (2008) 50:751–66. 10.1002/dev.20344 18814182 PMC2860183

[120] Kozlowska K Walker P McLean L Carrive P. Fear and the defense cascade: clinical implications and management. Harvard Rev Psychiatry (2014) 23:263–87. 10.1097/HRP.0000000000000065 26062169 PMC4495877

[121] Hagenaars MA Stins JF Roelofs K. Aversive life events enhance human freezing responses. J Exp Psychol. (2012) 141:98–105. 10.1037/a0024211 21767043

[122] Kozlowska K Palmer DM Brown KJ Scher S Chudleigh C Davies F. Conversion disorder in children and adolescents: a disorder of cognitive control. J Neuropsychol. (2015) 9:87–108. 10.1111/jnp.12037 24405496

[123] van der Kruijs SJM Vonck KEJ Langereis GR Feijs LMG Bodde NMG Lazeron RHC Autonomic nervous system functioning associated with psychogenic nonepileptic seizures: analysis of heart rate variability. Epilepsy Behav. (2016) 54:14–9. 10.1016/j.yebeh.2015.10.014 26615481

[124] Bowlby J. The nature of the child's tie to his mother. Int J Psychoanal. (1958) 39:350–73. 13610508

[125] Taylor DC. Hysteria, play-acting and courage. Br J Psychiatry (1986) 149:37–41. 10.1192/bjp.149.1.37 3779303

[126] Hohwy J Delusions, illusions and inference under uncertainty. Mind Lang. (2013) 28:57–71. 10.1111/mila.12008

